# Retraction Note: Antidiabetic, renal/hepatic/pancreas/cardiac protective and antioxidant potential of methanol/dichloromethane extract of *Albizzia Lebbeck Benth*. Stem bark (ALEx) on streptozotocin induced diabetic rats

**DOI:** 10.1186/s12906-023-04092-8

**Published:** 2023-07-15

**Authors:** Danish Ahmed, Vikas Kumar, Amita Verma, Pushpraj S Gupta, Hemant Kumar, Vishal Dhingra, Vatsala Mishra, Manju Sharma

**Affiliations:** 1 Department of Pharmaceutical Sciences, Faculty of Health Sciences, Sam Higginbottom Institute of Agriculture, Technology & Sciences, Deemed University, Allahabad, Uttar Pradesh India; 2 Department of Pharmacology, Faculty of Pharmacy, Jamia Hamdard, New Delhi, India; 3 Department of Pharmacology, Hamdard Institute of Medical Sciences & Research, Jamia Hamdard, New Delhi, India; 4 Department of Pathology, Moti Lal Nehru Medical College, Allahabad, India

**Retraction Note: BMC Complement Med Ther 14, 243 (2014)**

10.1186/1472-6882-14-243

The Editor has retracted this article. Concerns were raised about a number of the images presented in Figure 2, Figure 10 and Figure 11. The authors provided raw data; however, as there were inconsistencies in these data the Editor no longer has confidence in the results and conclusions presented. In addition, the authors have not provided evidence of appropriate ethical oversight of this study. Vikas Kumar disagrees with this retraction. Danish Ahmed, Amita Verma, Girja Shankar Shukla and Manju Sharma have not responded to correspondence from the Editor about this retraction.

